# The Influence of Cu-Additions on the Microstructure, Mechanical and Magnetic Properties of MnAl-C Alloys

**DOI:** 10.1038/s41598-020-64697-8

**Published:** 2020-05-12

**Authors:** Florian Jürries, Jens Freudenberger, Kornelius Nielsch, Thomas George Woodcock

**Affiliations:** 1 Leibniz IFW Dresden, Helmholtzstrasse 20, 01069 Dresden, Germany; 2 TU Dresden, Institute of Materials Science, 01062 Dresden, Germany; 3 TU Bergakademie Freiberg, Institute of Materials Science, Gustav-Zeuner-Str. 5, 09599 Freiberg, Germany

**Keywords:** Magnetic properties and materials, Materials science, Condensed-matter physics, Ferromagnetism

## Abstract

Alloys of the form (Mn_54_Al_44_C_2_)_100-x_Cu_x_ (with x = 0, 1, 2, 4 and 6) were produced by induction melting. After homogenisation and quenching, most of the alloys consist entirely of the retained ε-phase, except for x = 6, in which the κ-phase was additionally present. After subsequent annealing, the alloys with x ≤ 2 consist entirely of a Cu-doped, ferromagnetic τ-phase, whereas the alloys with x > 2 additionally contain the κ-phase. The polarisation of the alloys at an applied field of 14 T decreases with increasing Cu-content, which is attributed i) to the dilution of the magnetic moment of the τ-phase unit cell by the Cu atoms, which do not carry a magnetic moment, and ii) at higher Cu-contents, to the formation of the κ-phase, which has a much lower polarisation than the τ-phase and therefore dilutes the net polarisation of the alloys. The Curie temperature was not affected by the Cu-additions. The stress needed to die-upset the alloys with x ≤ 2 was similar to that of the undoped alloy, whereas it was much lower for x = 4 and 6, due to the presence of intergranular layers of the κ-phase. The extrinsic magnetic properties of alloys with x ≤ 2 were improved by die-upsetting, whereas decomposition of the τ-phase during processing had a deleterious effect on the magnetic properties for higher Cu-additions.

## Introduction

The growth of environmentally-friendly technologies such as wind power and electromobility has led to an increasing demand for high-performance permanent magnets such as those based on Nd_2_Fe_14_B^1,2^. Since such magnets contain significant amounts of rare-earth (RE) elements, the supply of which is described as being critical^3,4^, the question of developing sustainable, RE-free alternatives is becoming increasingly important^5^. RE-free permanent magnets based on Mn-Al-C have promising magnetic properties which originate from the metastable L1_0_-structured τ-phase^6^. It has been shown^7^ that anisotropic permanent magnets can be produced from MnAl-C alloys via hot extrusion, resulting in a maximum energy product of (BH)_max_ = 55 kJ/m^−3^. Although the non-critical nature^8^ and low cost of the raw materials and the magnetic properties make MnAl-C magnets highly attractive for applications, the production costs involved in the hot extrusion process are thought to be high^9^. A significant part of the production costs of hot extrusion originates from the tool wear^9^, which results in low lifetimes for the tools. An estimation for the lifetimes of extrusion tools was made by Krumphals *et al*.^10^ showing the following relationship:

1 $$\frac{dD}{dt} \sim {\left(\frac{{\sigma }_{eq}}{A}\right)}^{{m}_{l}}$$

with $\frac{{dD}}{{dt}}$ describing the damage rate, ${\sigma }_{{eq}}$ as the von Mises stress which corresponds to the flow stress of the material, while $A$ and ${m}_{l}$ are material and process dependent parameters. For extrusion ${m}_{l}\ge 1$^10,11^ and Eq. (1) shows that even a small reduction of the stress required results in a large reduction of the damage rate and hence increase the lifetime of the extrusion tools. Another estimation, given by Kojima *et al*.^12^, is that a 10% reduction of the stress applied for deformation results in a tenfold increase in the lifetime of the die.

It has been reported that the flow stress of MnAl-C materials can be reduced without degrading the magnetic properties by adding up to 2–3 at% of Cu^12^. Although that work shows a desirable effect, the role of Cu and its influence on the microstructure have not been studied in detail and with this knowledge, further optimisation may be possible.

The aim of this work is therefore to investigate the impact of Cu-additions on the microstructure, mechanical and magnetic properties of MnAl-C alloys. To this end, a series of MnAl-C-Cu alloys has been prepared, processed using die upsetting and characterised using a variety of techniques.

## Experimental

Different alloys with the quasi-binary composition (Mn_54_Al_44_C_2_)_100-x_Cu_x_ (x = 0, 1, 2, 4 and 6) were cast by induction melting. The elements used had a purity of 99.99% for Mn, Al and Cu (MaTeck GmbH), and 99.9% for carbon (GoodFellow GmbH). The as-cast rods were subjected to a two-step heat treatment, comprising 1) homogenisation at 1100 °C for 2 days followed by quenching into water and 2) annealing at 720 °C for 10 min, again followed by quenching in water. This annealing temperature of 720 °C was chosen to match the deformation temperature in the patent of Kojima *et al*.^12^.

The composition of the rods produced was quantified using Inductively Coupled Plasma Optical Emission Spectroscopy (ICP-OES, 0.2–1% relative standard derivation) for the metallic components and carrier-gas hot-extraction (0.5–5% relative standard derivation), which is suitable for analysing light elements, for the C. The results of these measurements are shown in Table 1. The difference between the measured and nominal compositions is thought to be due to the evaporation losses during the melting process.

**Table 1:** Measured compositions of the investigated alloys.

Alloy	Mn (at%)	Al (at%)	C (at%)	Cu (at%)
Mn_54_Al_44_C_2_	53.02	44.86	2.12	—
(Mn_54_Al_44_C_2_)_99_Cu_1_	52.58	44.11	2.22	1.10
(Mn_54_Al_44_C_2_)_98_Cu_2_	52.32	43.51	2.01	2.16
(Mn_54_Al_44_C_2_)_96_Cu_4_	51.40	42.36	1.98	4.26
(Mn_54_Al_44_C_2_)_94_Cu_6_	49.65	42.02	2.00	6.33

Sections of the annealed rods (10 mm height with 10 mm diameter) were deformed by die upsetting with a deformation rate of 0.001 s^−1^ at 720 °C in argon atmosphere. Heating from room temperature to the deformation temperature was realised by optical heating and took around 220 s with a non-linear heating rate. To reduce friction between punches and die, the punches were covered by a graphite solution. The samples were deformed to different logarithmic degrees of deformation, φ, to see the influence of this on the magnetic and structural properties. For more accurate measurement of the deformation stress, mechanical tests were done in compression on an electromechanical Instron 8562 device with a compression speed of *l*_0_ × 10^−3^ mms^−1^ at 720 °C, where *l*_0_ is the initial size of the sample. The samples used for this test were cut from the annealed rods using spark erosion and had a height of 8 mm with a diameter of 4 mm. As any possible pores and cracks in the samples would have a large influence on the measured mechanical properties, computed X-ray tomography was used to examine the internal state of the samples before the mechanical tests. The images were taken with a voxel size of vx = 4.5 µm³ and reconstruction of the 3D images was done using Phoenix datosjx2 software^13^.

For Differential Scanning Calorimetry (DSC) a Setaram Sensys Eco device was used to investigate the phase transitions of the quenched samples. The bulk samples were crushed to powder for this analysis. The measurements were done using a heating rate of 5 Kmin^−1^ in the temperature range from 30 °C to 830 °C in a 1 bar Ar-atmosphere.

The magnetic properties of the samples were investigated with a Quantum Design PPMS using the vibrating sample magnetometer (VSM) attachment to record M-H hysteresis loops up to an applied field of 14 T at room temperature. By measuring the deformed samples with magnetic field parallel and perpendicular to the pressing direction, the texture of the samples could be determined. Furthermore, thermomagnetic measurements were done at an applied field of 0.1 T in the temperature range from room-temperature to 440 °C. The measurements were carried out on spark eroded samples with a geometry of 2 × 2 × 1 mm, where the 1 mm dimension for the deformed samples was parallel to the direction of deformation. All magnetic measurements consider the demagnetisation factor $D$ as suggested by Aharoni^14^. For the used geometry this results in $D=\frac{1}{2}$ and $D=\frac{1}{4}$ for axial and in plane direction, respectively.

X-ray diffraction (XRD) measurements were carried out using bulk samples with a Bruker diffractometer using Co-K_α_ radiation (with λ(K_α1_) = 1.789007 Å and λ(K_α2_) = 1.792892 Å). To analyse the diffraction patterns Rietveld refinement with FullProf Suite software^15^ was used. The R_wp_ value was used as a measure of the goodness of fit and it lies below 1.8 for all fits. Microstructural analysis was done with the same samples using a Gemini Leo 1530 Field Emission Gun Scanning Electron microscope (SEM) after preparing the sample surface using standard metallographic techniques.

## Results and Discussion

### Structural analysis of undeformed samples

**Figure 1:**
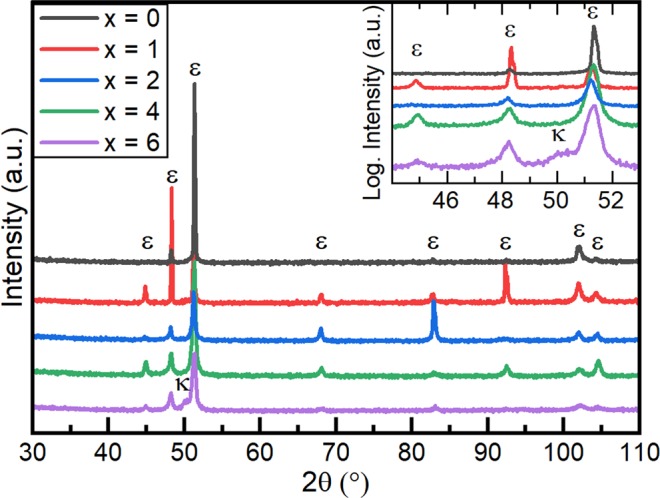
XRD patterns of the (Mn_54_Al_44_C_2_)_100-x_Cu_x_ alloys in the starting state i.e. after homogenisation and quenching.

**Figure 2:**
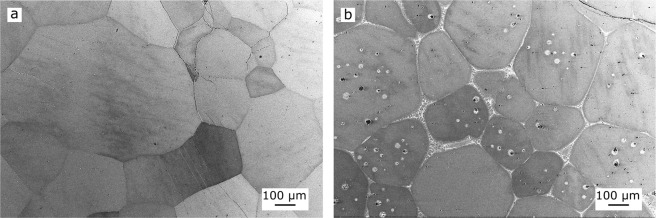
BSE images of homogenised and quenched (Mn_54_Al_44_C_2_)_100-x_Cu_x_ alloys, with (**a**) x = 1 and (**b**) x = 6.

The initial state of the samples after homogenisation and quenching is shown by the XRD results in Fig. 1 and the backscattered electron (BSE) images in Fig. 2. After homogenisation and quenching, all samples, except for x = 6, only showed XRD peaks corresponding to the ε-phase (P6_3_/mmc, Mg-type) (Fig. 1). The sample with x = 6 showed the presence of a small fraction of a second phase, κ (Pm $\bar{3}$ m, CsCl-type), in addition to ε-phase. The ε-phase was retained to room temperature via quenching. The microstructure of the samples was found to consist of equiaxed ε grains of the order of 100 µm in size (Fig. 2). In the case of the sample with x = 6, the additional κ-phase was observed as intergranular layers and as spherical intragranular precipitates (bright regions in Fig. 2b). As the ε grains in the homogenised materials are rather coarse and do not have a preferred orientation, the varied intensities of the ε peaks in the XRD patterns (Fig. 1) can be explained by different crystallographic orientations being sampled during the measurement of each alloy.

**Figure 3:**
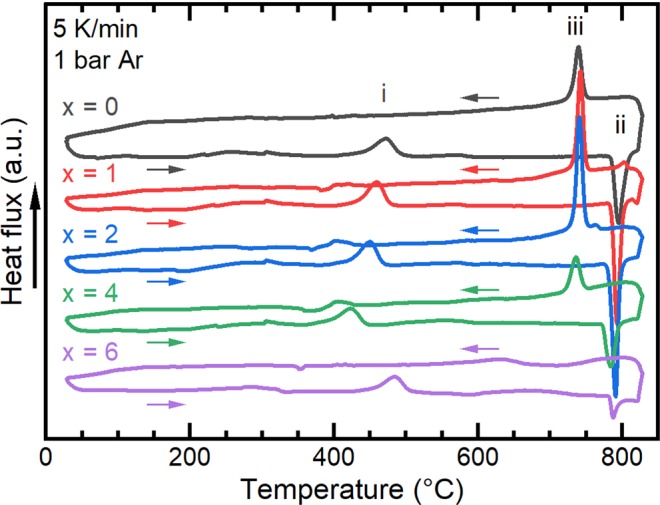
DSC curves from 30 °C to 830 °C of homogenised and quenched (Mn_54_Al_44_C_2_)_100-x_Cu_x_ alloys.

To see whether the Cu-addition has an influence on the phase transition temperature and in order to be able to choose appropriate heat treatment conditions to produce the τ-phase, DSC measurements were carried out on homogenised samples (Fig. 3). In Fig. 3, several peaks corresponding to phase transitions in the alloys are visible. While heating the homogenised and quenched samples from room temperature, the transformation from the quenched-in high temperature ε-phase into the metastable τ-phase starts at around 450 °C for the Cu-free alloy (i). When heating further, the metastable τ-phase transforms back to the high temperature ε-phase at around 800 °C (ii). When cooling the sample, the high temperature phase transforms back into τ-phase at around 750 °C (iii).

With increasing Cu-content up to 4 at%, the ε $\to$ τ phase transition shifts to a lower onset temperature, down to 393 °C. For the alloy with a Cu-content of 6 at% an increase of the transformation temperature up to 457 °C was observed. This correlates with the presence of intergranular layers of the second phase, κ, in the material before the DSC measurement (see Figs. 1 and 2b). Since the formation of the τ-phase via the massive mode starts preferentially at ε-ε grain boundaries^16–18^, the presence of the κ-phase at those boundaries may hinder the massive transformation and therefore lead to the increase of the ε $\to$ τ transformation temperature (i) shown in Fig. 3 for the alloy containing 6 at% Cu. The τ $\to$ ε transition temperature (ii) is less affected by the Cu-addition and changes only around 10 °C for the alloys investigated. It is noticeable that the integrated peak areas of the τ $\to$ ε phase transition (ii) and the transition of ε-phase when cooling (iii) are smaller for Cu-rich alloys (x $\ge$ 4 at%) compared to those of the alloys with lower Cu-content. One possible explanation is that for Cu-rich alloys, parts of the ε-phase transform into κ-phase during heating in the DSC experiment, thus reducing the volume fraction available for the transitions (ii) and (iii).

**Figure 4:**
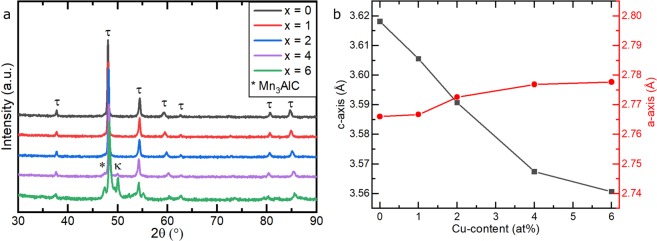
(**a**) XRD patterns of (Mn_54_Al_44_C_2_)_100-x_Cu_x_ alloys after annealing (720 °C for 10 min) and (**b**) the lattice parameters c (black squares) and a (red circles) of the τ-phase calculated from Rietveld refinement of the XRD data.

The homogenised and quenched samples were then annealed at 720 °C for 10 min with the aim of producing the ferromagnetic τ-phase as a single phase in all samples. Since the homogenised alloy with 6 at% Cu already showed a second phase next to the ε-phase it is expected that it will not be possible to get single-phase τ for this alloy. The structure of the annealed samples was analysed by XRD and SEM. The XRD patterns are shown in Fig. 4a. Rietveld refinement of these was used to calculate the lattice parameters of the τ-phase (Fig. 4b).

The XRD patterns show that for the alloys containing up to 2 at% of Cu only peaks of the τ-phase occur. For higher Cu content, additional peaks emerge, which are attributed to the κ-phase and also to Mn_3_AlC (Pm $\bar{3}$ m, CaTiO_3_-type). The lattice parameters of the τ-phase (Fig. 4b) refer to the primitive, *tP*2 representation of the tetragonal L1_0_ unit cell. With increasing Cu-content, the *c*-axis (black points in Fig. 4b) of the unit cell decreases in length quite strongly, while the *a*-axis (red) increases in length by a smaller amount. The dependence of the lattice parameter on the Cu-content in single-phase τ alloys shows that the Cu was dissolved in the τ unit cell.

### Magnetic properties of annealed samples

**Figure 5:**
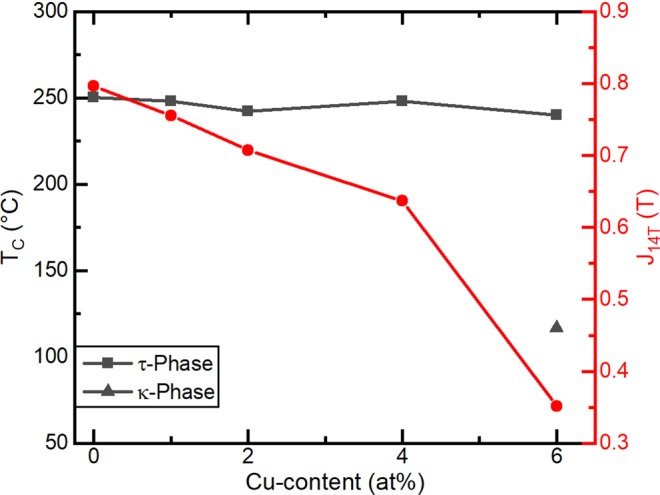
Curie temperature, T_C_, of the τ-phase (black squares), κ-phase (black triangle) and polarisation (red circles) of the alloy at an applied field of 14 T, J_14T_, as a function of Cu-content for (Mn_54_Al_44_C_2_)_100-x_Cu_x_.

The annealed samples were used to investigate the influence of Cu-addition on the Curie temperature, T_C_, and polarisation at an applied field of 14 T, J_14T_ (Fig. 5).

Given that T_C_ is normally very sensitive to the composition and that Cu is dissolved in the τ unit cell, it is surprising that there is no dependence of T_C_ of the τ-phase on the Cu-content visible from Fig. 5. *Sugihara et al*.^19^ reported a decrease of the T_C_ when partially substituting Mn with Cu in ternary Mn-Al-Cu alloys; however, such substitutions also decrease the Mn:Al ratio, which is known to lead to a reduced T_C_^20^. In the current work, the compositions of the alloys were chosen so that the Mn:Al ratio is constant (see Table 1), which may be the reason why little dependence on the Cu-content was observed. When comparing the T_C_ of alloys with similar Cu-contents from the literature^19^ and this work, the difference is mainly due to the fact that the materials in this work contain carbon, which has a large negative influence on T_C_^20^. For x = 6, an additional, lower T_C_ has been observed which is attributed to the κ-phase. Compared to the results of Sugihara *et al*.^19^, the κ-phase occurs here at lower Cu-content due to different heat treatment and alloy composition.

In Fig. 5, the influence of Cu-addition on the J_14T_ of the alloys is also shown. With increasing Cu-addition up to x = 4, the J_14T_ of the samples decreases, which can be explained by a dilution of the magnetic moment of the τ unit cell via the replacement of Mn atoms which have a high magnetic moment with Cu atoms, without a magnetic moment. The sample at x = 4, contains a negligible volume fraction of the κ-phase and therefore its J_14T_ follows the trend of the alloys with lower Cu content, which do not contain κ-phase. In contrast, at 6 at% Cu, J_14T_ decreases rapidly due to the formation of a larger volume fraction of the κ-phase, which has a lower saturation magnetisation than the τ-phase^21^.

### Effects of Cu-additions on deformation

**Figure 6:**
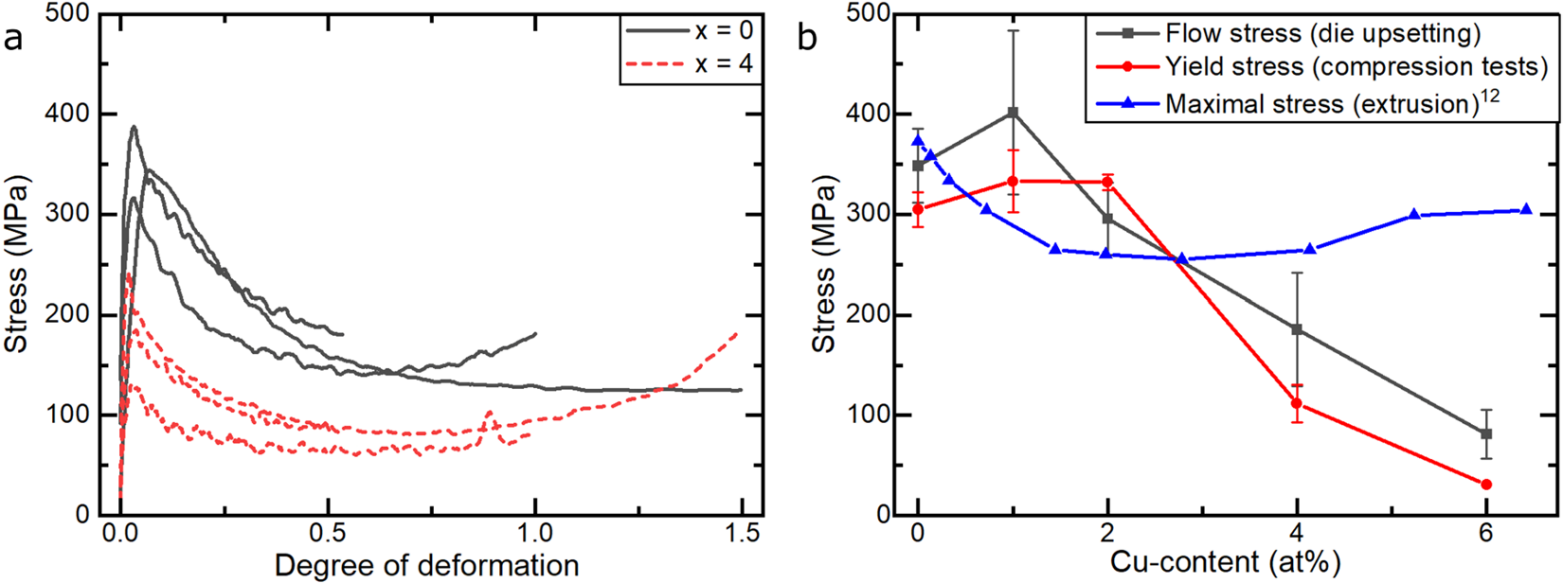
Deformation behaviour of (Mn_54_A_l44_C_2_)_100-x_Cu_x_ alloys deformed by die upsetting with 0.001 s^−1^ at 720 °C, (**a**) flow stress curve for different alloys, (**b**) peak stress as a function of Cu-Content; black and red: this work; blue: data from Kojima *et al*.^12^.

To investigate the influence of Cu-additions on the deformability of these alloys, the annealed samples were subjected to warm die-upsetting. In Fig. 6a, the deformation curves of two different alloys (x = 0, 4) deformed to three different logarithmic degrees of deformation (φ = 0.5, 1.0 and 1.5) are shown. Both alloys show similar behaviour: in the beginning of the compression, a rapid increase of the stress can be seen, which is followed by a maximum. After the maximum, the stress values decrease, which indicates the occurrence of dynamic recrystallization. The maximum can be found at a φ $\approx$ 0.03 for both alloys. The average values are 350 MPa and 185 MPa for the alloy with x = 0 at% and x = 4 at%, respectively. Three different deformation curves from the die-upsetting experiments per alloy were averaged, to show the influence of Cu-addition on the maximum stress (black points in Fig. 6b). In order to obtain more accurate values than those obtained during die-upsetting, compression tests were carried out at elevated temperature under controlled conditions. Similarly to the die-upsetting experiments, results of three compression tests per alloy were averaged and are shown in Fig. 6b as red points. The geometry of the samples for the compression tests was defined (see Experimental) and further control was added by inspecting the internal state of the samples using 3D computed X-ray tomography and rejecting those samples with significant internal flaws for the compression tests.

**Figure 7:**
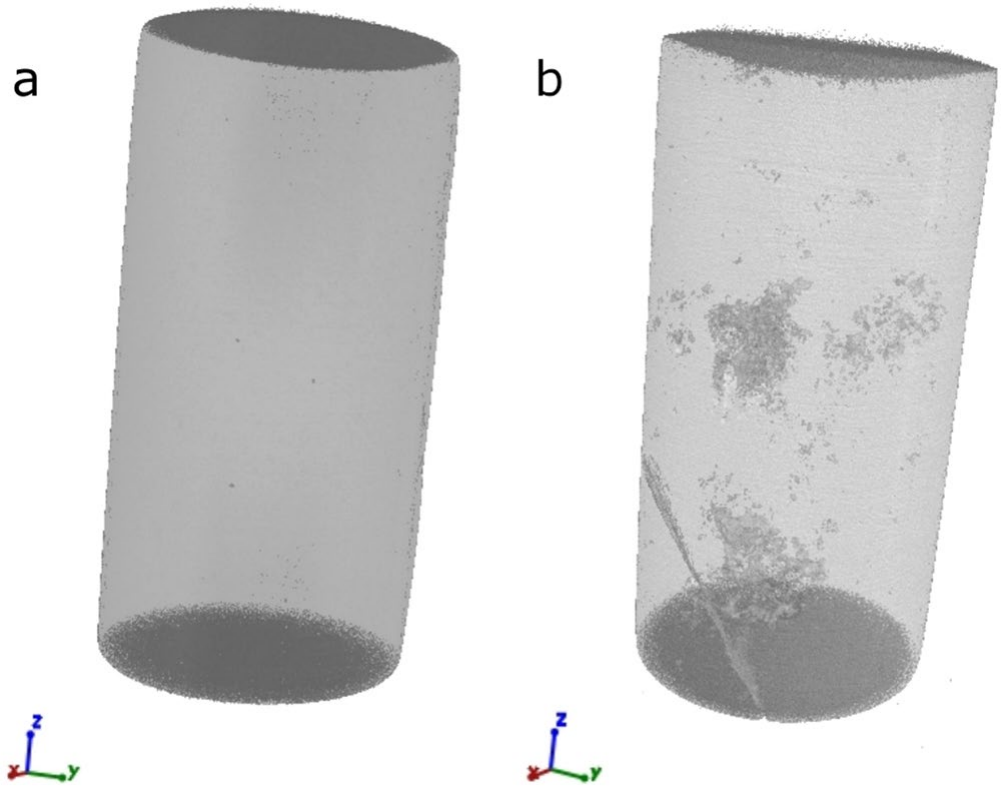
Computer tomographic 3D-images of two different samples for deformation tests. The samples show a different amount of pores and cracks (**a**) none visible, used for testing; (**b**) big cracks and pores are visible, not used for testing.

3D-images from a high-quality sample and from a sample containing internal flaws are shown in Fig. 7 as an illustration of the selection process. The additional analysis of the internal structure of the samples combined with the better defined experimental set-up results in more reliable values with smaller error bars (Fig. 6b).

Compared to the values given by Kojima *et al*.^12^, which are shown as blue triangles in Fig. 6b, our experiments led to a different behaviour of the maximum stress as a function of Cu-addition. Our experiments show a plateau-like behaviour at around 300–330 MPa for a Cu-content of up to 2 at%. Larger additions of Cu lead to a sharp decrease of the maximum stress down to 110 MPa and 30 MPa for 4 at% and 6 at% Cu, respectively. This is likely to be due to the presence of the κ-phase as intergranular layers in the Cu-rich alloys. Contrary to that, Kojima *et al*. observed a decrease of the maximum stress with increasing Cu-content, resulting in a minimum of 250 MPa at around 3 at% of Cu, followed by an increase to 300 MPa when alloying up to more than 6 at% of Cu. The reason for this difference is currently unknown.

### Microstructure and magnetic properties of die-upset samples

**Figure 8:**
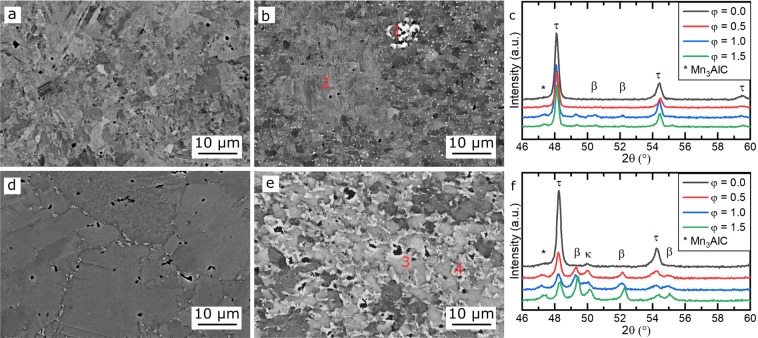
Annealed (Mn_54_Al_44_C_2_)_100-x_Cu_x_ alloys before and after deformation to φ = 1.5. (**a**) BSE image for x = 0, φ = 0, (**b**) BSE image for x = 0, φ = 1.5, (**c**) XRD patterns for x = 0 in different deformation states, (**d**) BSE image for x = 4, φ = 0, (**e**) BSE image for x = 4, φ = 1.5, (**f**) XRD patterns for x = 4 in different deformation states.

The (Mn_54_Al_44_C_2_)_100-x_Cu_x_ alloys investigated in this work can be classified into two groups with different microstructures: low-Cu alloys (x ≤ 2 at% Cu) and Cu-rich alloys (x > 2 at% Cu). The alloys with x = 0 and x = 4, shown in Fig. 8, are representative of these two groups. Figure 8(a,d), show the microstructure in the undeformed state for the x = 0 and x = 4, whereas Fig. 8(b,e) show the microstructure in the deformed state with φ = 1.5 for the same alloys. The XRD patterns for all the different deformation states studied are given in Fig. 8(c,f) for x = 0 and x = 4, respectively.

As seen from the XRD data in Fig. 8(c,f), multiple phases occur in these alloys. The Mn_3_AlC phase can be identified in the SEM as small needle-like precipitates at higher resolution (not shown here). As the Mn_3_AlC precipitates occur in deformed samples of both groups of alloys in a similar phase fraction, they are not considered to contribute to differences in the behaviour of the two groups and will not be considered further here.

Starting in the annealed state, shown in Fig. 8a, the microstructure of the low-Cu alloys is confirmed to consist of a single τ-phase. With ongoing hot deformation, the decomposition of the metastable τ-phase begins, which results in β-Mn precipitates. In Fig. 8b, these are visible as bright spots which sometimes form clusters (marked “1” in Fig. 8b) but are mostly spread along the grain boundaries of the recrystallized τ-phase. In the areas of the bigger non-recrystallised grains (marked “2” in Fig. 8b) no precipitates of β-Mn are visible.

In contrast, the annealed state of the Cu-rich alloys (Fig. 8d) already shows some bright precipitates along prior ε grain boundaries, indicating a second phase. These can be identified as κ-phase using the XRD patterns, shown in Fig. 8f. In the deformed samples, the decomposition of the metastable τ-phase appears to be more rapid in these Cu-rich alloys than in the low-Cu alloys, and thus a larger fraction of β-Mn and κ-phase precipitates is observed. Both of these phases appear bright in the BSE images, but far less Cu can be dissolved in β-Mn than in the κ-phase^22^ and therefore they can be distinguished using EDX measurements. The Cu-rich κ-phase corresponds to the regions marked “3” in Fig. 8e, while the Cu-poor β-Mn is marked “4” in the same image. In Table 2, the different phase fractions in the undeformed (φ = 0) and deformed (φ = 1.5) states are compiled. These were calculated by Rietveld refinement of the XRD-patterns (Fig. 8(c,f)) and an error of ± 0.1 can be assumed, which results partly from the XRD measurement and partly from the refinement. For both alloys in the undeformed state, the fraction of the τ-phase is close to 1. After the deformation, part of the τ-phase decomposed into β-Mn and Mn_3_AlC, but still around 0.90 of τ-phase remained for x = 0. In contrast, there is less than 0.50 of the τ-phase remaining for x = 4 and much more β-Mn is visible together with an increased content of κ-phase. The values shown here support the idea that in alloys that contain the κ-phase, the decomposition of the metastable τ-phase is accelerated, which is also shown in the SEM-images in Fig. 8.

**Table 2:** Weight fraction of different phases for x = 0 and x = 4 before (φ = 0) and after deformation (φ = 1.5).

Alloys	τ-phase	β-Mn	Mn_3_AlC	κ-phase
Mn_54_Al_44_C_2_ φ = 0	1.00	0.00	0.00	0.00
(Mn_54_Al_44_C_2_)_96_Cu_4_ φ = 0	0.99	0.00	0.00	0.01
Mn_54_Al_44_C_2_ φ = 1.5	0.91	0.04	0.05	0.00
(Mn_54_Al_44_C_2_)_96_Cu_4_ φ = 1.5	0.35	0.52	0.06	0.07

**Figure 9:**
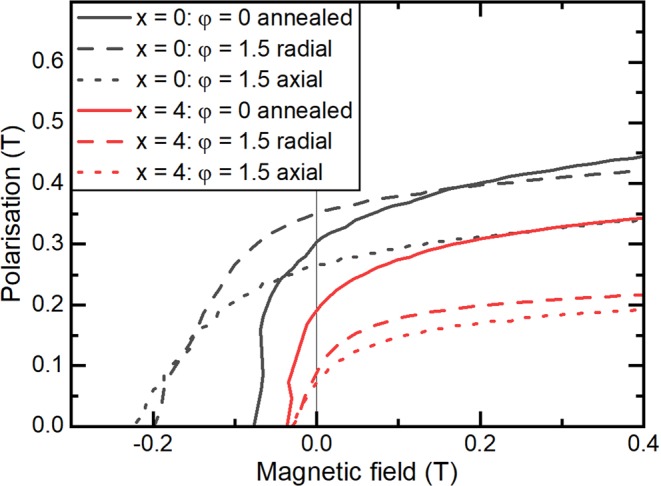
Demagnetisation curves of (Mn_54_Al_44_C_2_)_100-x_Cu_x_ alloys with x = 0 (black lines) and x = 4 (red lines) measured before and after hot deformation to φ = 1.5. The deformed samples were measured with the external field applied parallel to the pressing direction (“axial”) and perpendicular to it (“radial”).

Because of their different microstructure, the low-Cu and Cu-rich alloys will show different magnetic properties as well. In Fig. 9, hysteresis loops of alloys with x = 0 at% Cu and x = 4 at% Cu of annealed and hot deformed samples are shown. Due to the reorientation of the grains during recrystallization, the deformed samples should show a texture where the magnetically easy <001> axis of the grains tends to be aligned perpendicular to the axis along which pressure was applied during die-upsetting. In order to observe this, hysteresis loops for deformed samples were carried out with the external field applied parallel and perpendicular to this pressing direction. To calculate the degree of texture ($\omega$) from the remanence measured in both sample orientations, following equation was used:

2 $$\omega =\frac{{J}_{R,radial}-{J}_{R,axial}}{{J}_{R,radial}}$$

where ${J}_{R}$ is the remanent polarisation, which was measured in the radial and axial directions of the sample, which are perpendicular and parallel to the pressing direction, respectively. At φ = 1.5, the degree of texture for the alloys with x = 0 and x = 4 was $\omega$ = 0.31 and $\omega$ = 0.33, respectively, which indicates that a weak texture was present.

As a result of the texture formed during deformation, the low-Cu alloys showed higher remanence when measured with the field applied perpendicular to the pressing direction (“radial” in Fig. 9) than when measured with the field applied parallel to the pressing direction (“axial” in Fig. 9). The remanence of the low-Cu, annealed materials was between these two values because the annealed materials were crystallographically isotropic. The degree of texture in the low-Cu materials is similar to that reported in the literature for die-upset Cu-free compositions^23^. In the case of the deformed Cu-rich alloys, although the remanence values are different in the two measurement directions, which suggests the formation of a texture, the absolute values of the remanence in the deformed material are much lower than in the annealed state. This reflects the significant decomposition of the τ-phase during the die-upsetting, which was shown by the BSE image in Fig. 8e.

**Figure 10:**
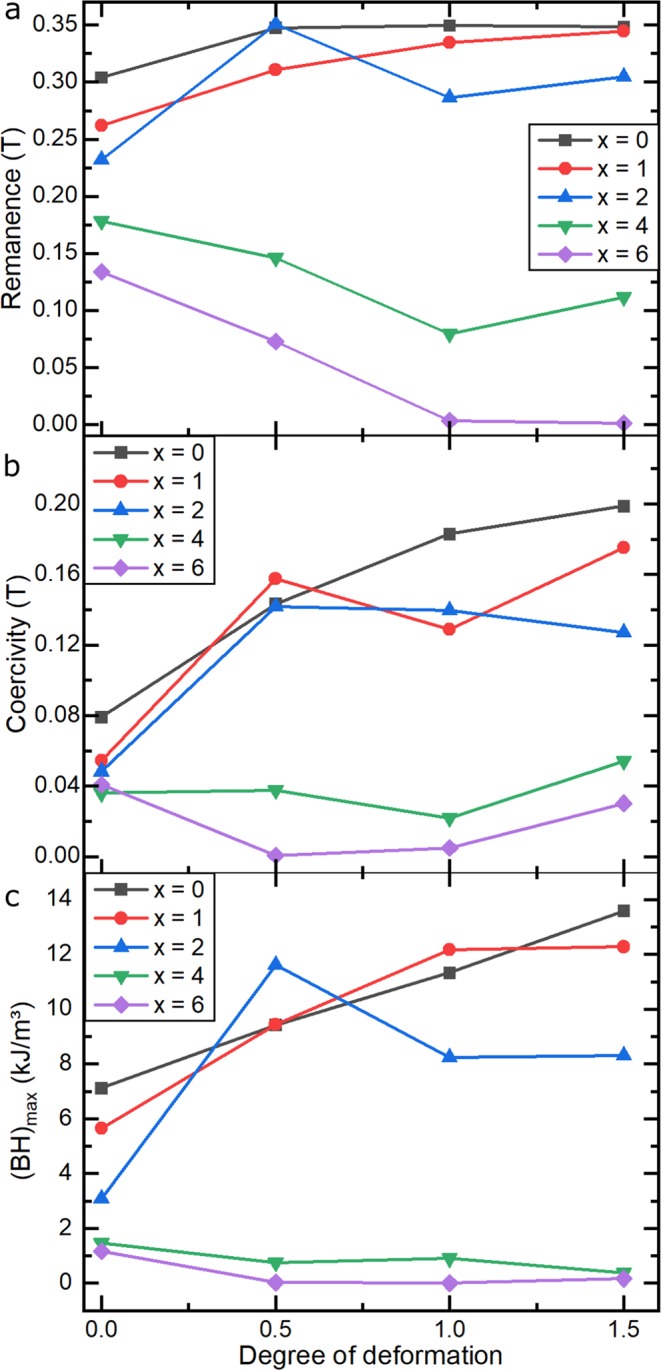
Magnetic properties (**a**) remanence, (**b**) coercivity and (**c**) (BH)_max_ of (Mn_54_Al_44_C_2_)_100-x_Cu_x_ alloys as a function of the degree of deformation.

In Fig. 10, the remanence (Fig. 10a), coercivity (Fig. 10b) and energy product, (BH)_max_ (Fig. 10c) of all the alloys are shown as a function of the logarithmic degree of deformation, φ. The values refer to measurements in the radial direction. The low-Cu alloys reveal an increase in all of these properties with increasing φ. The increase in remanence for low-Cu alloys emerges from the formation of a planar texture during the hot deformation, whereas the increased coercivity is connected to the grain refinement during dynamic recrystallisation and as a result, an increased pinning of domain walls at the grain boundaries^24^. The grain refinement mainly takes place at φ < 0.5, which is why the increase of remanence as a function of φ tends to flatten off. Due to the changes in the microstructure, the low-Cu alloys of this group exhibit higher (BH)_max_ values in the deformed state. Contrary to that, the Cu-rich alloys show no increase or even a slight decrease of the extrinsic properties, which is connected to the more rapid decomposition of the τ-phase into β-Mn and to the formation of the κ-phase.

## Conclusions

In the current work, the effects of Cu-additions on the microstructure, mechanical and magnetic properties of (Mn_54_Al_44_C_2_)_100-x_Cu_x_ alloys in various states have been investigated. It was shown that Cu can be dissolved in the ferromagnetic τ-phase up to the solubility limit, which lies between 2 at% Cu and 4 at% Cu for the compositions studied here. The dissolved Cu shortens the *c*-axis of the L1_0_*-*structured unit cell of the τ-phase, while increasing the length of the *a*-axis. In alloys with Cu-additions of 4 at% or more, the κ-phase formed in addition to the τ-phase. The alloys were therefore grouped into low-Cu (x ≤ 2) alloys, in which the κ-phase was not observed, and Cu-rich (x > 2) alloys in which the κ-phase formed. The polarisation at an applied field of 14 T decreased with increasing Cu-addition due to the dilution effect for the low-Cu alloys, and due to the formation of the κ-phase for Cu-rich alloys. The Curie temperature (T_C_) of around 250 °C for the τ-phase was surprisingly not affected by the addition of Cu, which may have been due to the constant Mn:Al ratio in the alloys studied here. A second T_C_ of around 120 °C occurs when the κ-phase is present. The stress needed to deform the low-Cu alloys did not depend strongly on their Cu-content; however, when the Cu-content was further increased to the level at which the κ-phase was present, the deformation stress decreased rapidly. This is thought to be due to the location of the κ-phase as intergranular layers in the microstructure. The low-Cu alloys showed enhanced magnetic properties as a result of the die upsetting, whereas the magnetic properties of the Cu-rich alloys were drastically degraded following die-upsetting as a result of the decomposition of the metastable τ-phase and the associated formation of the β-Mn and κ phases with inferior magnetic properties. Although the effect of Cu-additions on the microstructure, magnetic and mechanical properties of die-upset MnAl-C alloys has been established here, the effect reported by Kojima *et al*.^12^, where the deformation stress was reduced by approx. 30% with Cu-additions up to 3 at% without significantly degrading the magnetic properties could not be reproduced. Further work will be required in order to ascertain whether Cu-additions can be used to reduce the potential production costs of MnAl-C magnets.

## Supplementary information

Supplementary information.
