# Supplementary material for: The Influence of Cu-Additions on the Microstructure, Mechanical and Magnetic Properties of MnAl-C Alloys
Source: Sci Rep. 2020 May 12;10:7897. doi: 10.1038/s41598-020-64697-8 (PMC7217857; doi:10.1038/s41598-020-64697-8)
Supplement: Supplementary file 1 — Supplementary information. [file 41598_2020_64697_MOESM1_ESM.pdf]

Supplementary Information

The Influence of Cu-Additions on the Microstructure, Mechanical and Magnetic Properties of MnAl-C Alloys

Florian Jürries[1,2*], Jens Freudenberger[1,3], Kornelius Nielsch[1,2] and Thomas George Woodcock[1]

[1]Leibniz IFW Dresden, Helmholtzstrasse 20, 01069 Dresden, Germany

[2]TU Dresden, Institute of Materials Science, 01062 Dresden, Germany

[3]TU Bergakademie Freiberg, Institute of Materials Science, Gustav-Zeuner-Str. 5, 09599 Freiberg, Germany

*Correspondance to f.juerries@ifw-dresden.de

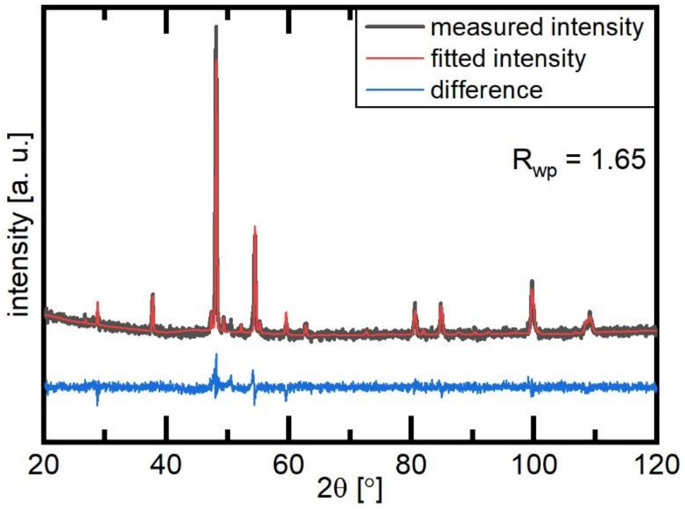

Supplementary 1: Used fitting profile of $Mn_{54}Al_{44}C_2$ after deformation to φ = 1.5. The R_{wp} value for this fit is 1.65.

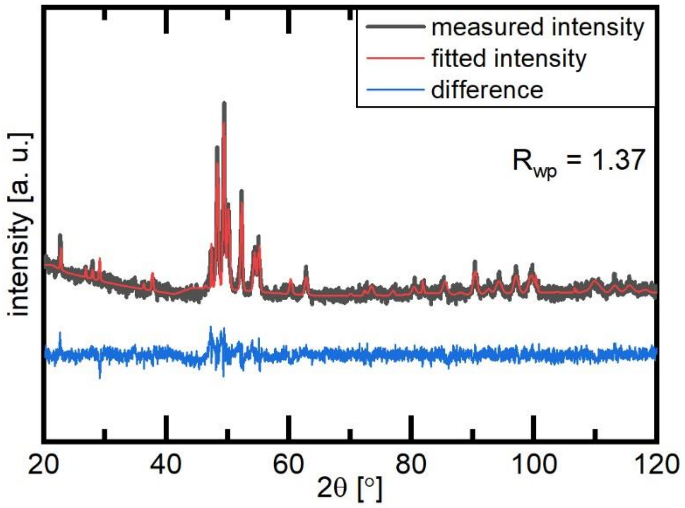

Supplementary 2: Used fitting profile of $(Mn_{54}Al_{44}C_2)_{96}Cu_4$ after deformation to φ = 1.5. The R_{wp} value for this fit is 1.37.
